# Safety and efficacy of neoadjuvant cisplatin + S-1 combined with radiation therapy for locally advanced non-small cell lung cancer

**DOI:** 10.1007/s00595-025-03019-9

**Published:** 2025-02-27

**Authors:** Takashi Karashima, Shinkichi Takamori, Miyuki Abe, Yohei Takumi, Atsushi Osoegawa, Kenji Sugio

**Affiliations:** Department of Thoracic and Breast Surgery, Faculty of Medicine, Oita University, 1-1 Idaigaoka, Hasama-Machi, Yufu, 879-5593 Japan

**Keywords:** Neoadjuvant therapy, Chemoradiotherapy, Non-small cell lung cancer, Cisplatin, S-1, Radiation

## Abstract

**Purpose:**

To assess the safety and efficacy of neoadjuvant chemoradiotherapy with cisplatin plus S-1 for advanced non-small cell lung cancer (NSCLC), with a focus on real-world outcomes.

**Methods:**

This retrospective study analyzed 32 patients with stage II-III NSCLC eligible for resection, who received preoperative induction therapy between January 2012 and December 2022. Specifically, 20 patients received cisplatin, S-1, and radiation therapy.

**Results:**

Among the 32 patients who received induction therapy, the objective response rate (ORR) was 56.2%, and surgical resection was feasible in 29 patients (90.6%). The 5 year recurrence-free survival (RFS) rate was 76.4%, and the 3- and 5 year overall survival (OS) rates were 86.2% and 82.3%, respectively. In the cisplatin + S-1 + radiation therapy group (n = 20), the ORR was 65.0%, and surgical resection was feasible in 17 patients (85.0%). The 3-year RFS and OS rates were 78.3% and 83.8%, respectively. Ef. 3 (complete pathological response) was observed in 3 patients (10.3%). No recurrences occurred in the non-adenocarcinoma subgroup (n = 6), indicating better outcomes relative to the adenocarcinoma group (5-year RFS, 100% vs. 61.4%; p = 0.07).

**Conclusions:**

Induction therapy, particularly with cisplatin + S-1 + radiation was associated with promising RFS and OS in locally advanced NSCLC, with favorable tolerability and effectiveness.

## Introduction

Lung cancer is one of the major causes of cancer-related death in most countries, and non-small cell lung cancer (NSCLC) accounts for approximately 85% of all cases [1]. Neoadjuvant chemoradiotherapy for locally advanced NSCLC has proven beneficial in multiple controlled trials. The chemotherapy regimens used in these trials included taxanes (paclitaxel and docetaxel), etoposide, or vinorelbine in combination with platinum-based drugs such as cisplatin and carboplatin [2–4]. This multimodal treatment strategy not only aims at preoperative tumor size reduction but also seeks to mitigate the risk of systemic spread by addressing micrometastatic disease, thereby potentially improving overall survival rates and quality of life for affected individuals. The evolving landscape of NSCLC treatment continues to explore the optimization of these therapeutic combinations and sequences to maximize patient benefits while minimizing associated toxicities.

The safety and efficacy of cisplatin + S-1 combined with radiation therapy have been reported in several randomized trials in patients with unresectable locally advanced NSCLC [5–8]. S-1 (TS-1; Taiho Pharmaceutical Co., Ltd, Tokyo, Japan) is a second-generation oral anticancer agent in the 5-FU series that incorporates a trio of active constituents: tegafur (a prodrug of 5-FU), gimeracil, and oteracil potassium. The antitumor efficacy of 5-FU is primarily mediated through the inhibition of the S phase within the DNA cycle, a mechanism that has been previously demonstrated to enhance the radiosensitivity of tumor cells [9, 10]. Furthermore, S-1 can be administered at its full dosage in conjunction with radiation therapy, showing significant therapeutic benefits with comparable progression-free survival (PFS) and overall survival (OS) and mild toxicities in the treatment of advanced NSCLC [5, 11]. To date, multiple reports have highlighted the efficacy of preoperative treatment regimens that combine cisplatin and S-1 + radiation therapy in advanced NSCLC. Recent studies, including those by Takamochi et al*.* [12], have demonstrated a high pathological complete response (pCR) rate in 43 cases of advanced squamous cell carcinoma, raising expectations for the potent antitumor effect of this combination therapy. However, the availability of real-world clinical data on the safety and efficacy of this treatment modality for resectable locally advanced NSCLC remains limited, with few comprehensive reports on this subject. Therefore, this retrospective study aimed to review real-world clinical data on preoperative adjuvant treatment with cisplatin + S-1 + radiation to determine its usefulness and safety, thereby expanding treatment options for advanced NSCLC and contributing to better treatment strategies.

## Methods

### Patients

We retrospectively identified 32 patients with potentially resectable stage II-III NSCLC who underwent preoperative induction therapy between January 2012 and December 2022 at the Department of Thoracic and Breast Surgery, Oita University Faculty of Medicine. Specifically, 20 patients treated with the combination of cisplatin + S-1 + radiation therapy were evaluated. The clinicopathological features of the patients, including age, sex, smoking status, tumor histology, clinical and pathological stages, treatment response, toxicities of induction therapy, surgical procedure, postoperative complication, complete resection rate, and driver oncogene mutation statuses (e.g. *epidermal growth factor receptor [EGFR]* and *c-ros oncogene 1[ROS1] rearrangement* and *anaplastic lymphoma kinase [ALK] rearrangement*) were examined. Regarding the staging modalities prior to induction therapy, all patients underwent contrast-enhanced computed tomography (CT), positron emission tomography-computed tomography (PET-CT), and brain magnetic resonance imaging (MRI). Post-induction therapy, contrast-enhanced CT and PET-CT were repeated to assess the therapeutic response radiographically. Routine brain MRI was not performed after induction therapy. The diagnosis of N2 prior to induction therapy is determined pathologically using endobronchial ultrasound-guided transbronchial needle aspiration (EBUS-TBNA) or transesophageal fine-needle aspiration (FNA) in cases where a tissue diagnosis is feasible, while in other cases, the diagnosis is based on imaging findings from contrast-enhanced CT and PET-CT. The clinical and pathological stages were defined based on the 8th edition of the American Joint Committee on Cancer lung cancer staging system [13]. Clinical information and follow-up data were obtained from patient medical records.

### Induction treatment

The indications for the combined use of cisplatin + S-1 + radiation therapy include: (1) patients with a good general condition, assessed as Performance Status (PS) 0–1, who are deemed capable of tolerating chemoradiotherapy; (2) absence of pulmonary diseases, such as interstitial pneumonia, which could be exacerbated by radiation; (3) a diagnosis of NSCLC confirmed through a biopsy of the primary lesion prior to the initiation of treatment; (4) advanced NSCLC determined by CT imaging to have hilar or mediastinal lymph node enlargement exceeding 10 mm in the short axis, suggestive of lymph node metastasis, yet considered resectable; and (5) cases where, despite being N0, there is involvement of the surrounding organs (e.g., chest wall invasion causing symptoms such as chest pain), where it is deemed more beneficial to reduce symptoms and achieve downstaging through preoperative treatment rather than proceeding directly to surgery. There are no age restrictions on eligibility for preoperative therapy, and it is administered to elderly patients as long as their overall health is good and their organ function is preserved. Surgery was performed in cases where, following neoadjuvant therapy, imaging evaluation showed no disease progression (PD) of the tumor, and the patients were deemed capable of undergoing surgery and suitable for complete resection.

The following section details the specifics of the cisplatin + S-1 + radiation therapy regimen. S-1 (40 mg/m^2^ twice a day [b.i.d.]) was administered orally in 2 separate doses from days 1–14 and days 22– 35. The dose of S-1 was determined based on the body surface area as follows: 80 mg/day for < 1.25 m^2^; 100 mg/day for 1.25–1.49 m^2^; and 120 mg/day for ≥ 1.5 m^2^. Cisplatin (60 mg/m^2^) was administered on days 1 and 22. We have generally used a 40 Gy dose for concurrent chemoradiation. Radiation therapy was started simultaneously with chemotherapy, with a total of 40 Gy delivered at 2 Gy once a day, 5 days a week. There have been no reports of extended survival periods with a higher dose of 60 Gy, and reports suggest an increase in adverse events (e.g., postoperative pneumonia with high-dose radiation therapy) [14]. In our study, only one patient with chest wall involvement received a high dose of radiation therapy (60 Gy), which was exclusively directed at chest wall invasion and primary lesions without targeting the lymph nodes, with regimens other than cisplatin + S-1 + radiation therapy, including carboplatin + paclitaxel + radiation therapy, other platinum-based combination therapies, and chemotherapy alone. Details of the preoperative treatment breakdown are listed in the “Results” section. Objective tumor responses were evaluated according to the Response Evaluation Criteria in Solid Tumors (version 1.1) [15]. Toxicities of induction therapy and postoperative complications were evaluated according to the Common Terminology Criteria for Adverse Events v5.0 (CTCAE) [16]. The end of the follow-up period was August 31, 2023.

### Surgery

After preoperative induction therapy, contrast-enhanced CT and PET-CT were performed, and surgery was conducted in cases where no new lesions or distant metastases were detected and radical resection was deemed possible. In all cases, the surgical procedure involves lobectomy or more extensive resection (e.g., bilobectomy or pneumonectomy in some cases), along with systematic lymph node dissection. At our institution, nearly all surgeries are performed via video-assisted thoracoscopic surgery (VATS) or open thoracotomy, rather than complete video-assisted thoracoscopic surgery (cVATS). This approach is used because of the increased risk of pulmonary artery injury following radiation to the pulmonary hilum and because cVATS has been reported to improve only short-term outcomes relative to thoracotomy [17]. To ensure safety, precise tumor resection, and thorough lymph node dissection, surgery via VATS or open thoracotomy is preferred.

### Adjuvant therapy

Postoperative adjuvant therapy is recommended for patients who have achieved R0 resection and are in sufficiently good health to undergo such treatment. Decisions regarding the administration of postoperative adjuvant therapy are not based on lymph node involvement or pathological stage; rather, it is generally applied to all patients who undergo surgery following preoperative therapy, and the regimen for adjuvant therapy generally replicates that used in induction therapy. However, modifications may be necessary based on the histological responses observed in the resected specimens. Specifically, for patients classified as Ef.0 or Ef.1, indicating a poor response to preoperative treatment with cisplatin + S-1 + radiation therapy, a different regimen (either cisplatin plus vinorelbine or cisplatin plus pemetrexed) may be considered. There were no prescribed imaging modalities during adjuvant therapy, and contrast-enhanced CT, PET-CT, and brain MRI were performed at the discretion of the treating physician. Additionally, there were no set intervals for imaging to monitor recurrence and imaging tests were performed when clinically indicated.

### Ethical information

This study was approved by the Institutional Review Board of the Oita University Faculty of Medicine (IRB No. 2412), and written informed consent was obtained from each patient.

#### PD-L1 tumor expression and EGFR mutation, ROS1 rearrangement and ALK rearrangement analyses

Immunohistochemical staining for programmed cell death ligand 1(PD-L1) was performed using the pharmDx antibody (Clone 22C3, Agilent Technologies Inc. [Dako], Santa Clara, CA, United States). *EGFR mutation* was determined by either CyCleave PCR (SRL Inc., Tokyo, Japan) or a KIT COBAS EGFR AMP/DET V2 24 T IVD (SRL Inc.). *ROS1 rearrangement* was determined by an AmoyDx^®^ ROS1 Gene Fusions Detection Kit (Amoy Diagnostics Co., Ltd. China). *ALK rearrangement* was performed using a VENTANA OptiView ALK kit (clone name D5F3) kit (Roche, Basel, Switzerland). The expression of PD-L1 and driver gene mutations are typically assessed in biopsy specimens obtained before induction therapy. However, if preoperative testing is not feasible owing to insufficient sample quantity or other issues, these assessments are performed on surgical specimens.

### Statistical analysis

Recurrence-free survival (RFS) was defined as the time from the initial treatment to clinical or radiographic progression or death, and OS was defined as the time from the initial treatment to the date of the last follow-up or death. The Kaplan–Meier method was used to estimate OS and RFS. P values of < 0.05 were considered to indicate statistical significance*.* All statistical analyses were performed using EZR version 1.62 [18].

## Results

### Patient characteristics

Table 1 details the characteristics of the 32 patients who underwent neoadjuvant induction therapy. These patients are divided into two groups: 20 received cisplatin + S-1 + radiation therapy, while 12 underwent alternative therapeutic modalities. The median age across all groups was 66 years. A majority of the patients were male (75.0%) and had a history of smoking (81.3%). Among these, 15 patients (46.9%) were diagnosed with adenocarcinoma and 10 (31.3%) with squamous cell carcinoma, with these frequencies being comparable in the cisplatin + S-1 + radiation therapy group and slightly lower in the other groups. The most common clinical stage was IIIA, observed in 18 patients (56.3%), with similar distributions in both treatment groups. A pathological diagnosis of N2 was performed in 9 cases using EBUS-TBNA or transesophageal FNA for mediastinal lymph node biopsy. In the remaining 23 cases, the presence or absence of lymph node metastasis was determined based on imaging findings from contrast-enhanced CT and PET-CT. Genetic aberrations encompassed *EGFR* mutations in 4 patients and *ROS1* alteration in 1 patient. There were no cases with A*LK translocation*. In the group receiving CDDP + S-1 + radiation therapy (n = 20), the high expression of PD-L1 (> 50%) was observed in just 1 of the 9 patients who were tested.

**Table 1:** Patient characteristics (n = 32) and surgical/pathological outcomes (n = 29)

Patient characteristics (n = 32)	All (n = 32)	CDDP + S-1 + RT (n = 20)	Others (n = 12)
Age (years)	66(38–76)	66(41–76)	65(38–70)
Sex, n(%)			
Male	24(75.0)	15(75.0)	9(75.0)
Female	8(25.0)	5(25.0)	3(25.0)
Smoking status, n(%)			
Never	6(18.8)	2(10.0)	4(33.4)
Ever	26(81.3)	18(90.0)	8(66.7)
Histology, n(%)			
Adenocarcinoma	15(46.9)	11(55.0)	4(33.4)
Squamous cell carcinoma	10(31.2)	6(30.0)	4(33.4)
Large cell carcinoma	2(6.3)	1(5.0)	1(8.3)
Adenosquamous carcinoma	2(6.3)	1(5.0)	1(8.3)
Others	3(9.4)	1(5.0)	2(16.7)
cTN, n(%)^a^			
cT2bN0	2(6.3)	0(0.0)	2(16.7)
cT1-2N1	4(12.5)	2(10.0)	2(16.7)
cT3N0	3(9.4)	3(15.0)	0(0.0)
cT3N1	2(6.3)	1(5.0)	1(8.3)
cT1-2N2	13(40.6)	9(45.0)	4(33.4)
cT3N2	3(9.4)	1(5.0)	2(16.7)
cT4N1	3(9.4)	2(10.0)	1(8.3)
cT4N2	2(6.3)	2(10.0)	0(0.0)
cStage, n(%)^a^			
IIA	2(6.3)	0(0.0)	2(16.7)
IIB	7(21.9)	5(25.0)	2(16.7)
IIIA	18(56.3)	12(60.0)	6(50.0)
IIIB	5(15.6)	3(15.0)	2(16.7)
Induction therapy, n(%)			
Cisplatin + S-1 + Radiation	20(62.5)	20(100.0)	0(0.0)
Carboplatin + Paclitaxel + Radiation	3(9.4)	0(0.0)	3(25.0)
Platinum-based doublet + Radiation	3(9.4)	0(0.0)	3(25.0)
Chemotherapy only	6(18.8)	0(0.0)	6(50.0)
Mutation type, n(%)			
* EGFR*^*c*^	4(12.5)	2(10.0)	2(16.7)
* ROS1*^*d*^	1(3.1)	1(5.0)	0(0.0)
Negative	19(59.4)	12(60.0)	7(58.3)
Not assessed	8(25.0)	5(25.0)	3(25.0)
PD‑L1^e^ expression, n (%)			
≥ 50%	4(12.5)	1(5.0)	3(25.0)
1–49%	10(31.3)	7(35.0)	3(25.0)
Negative	3(9.4)	1(5.0)	2(16.7)
Not assessed	15(46.9)	11(55.0)	4(33.4)
Objective response, n(%)			
Partial response	18(56.3)	13(65.0)	5(41.7)
Stable disease	13(40.6)	7(35.0)	6(50.0) 89
Progressive disease	1(3.1)	0(0.0)	1(8.3)
Surgical/ pathological outcomes(n = 29)	All (n = 29)	CDDP + S-1 + RT (n = 17)	Others (n = 12)
Procedure, n(%)			
Pneumonectomy	5(17.2) lobectomy	2(11.8)	3(25.0)
Lobectomy^b^	24(82.8)	15(88.2)	9(75.0)
Combined resection, n(%)			
Chest wall	3(10.3)	3(17.6)	0(0.0)
Pulmonary artery	1(3.4)	1(5.9)	0(0.0)
Resection rate, n(%)			
R0	26(89.7)	15(88.2)	11(91.7)
R1(malignant pleural effusion)	2(6.9)	1(5.9)	1(8.3)
R1(pleural fluid cytology)	1(3.4) Lobectomy	1(5.9)	0(0.0)
Histologic response, n(%)			
Ef.1	14(48.3)	6(35.3)	8(66.7)
Ef.2	10(34.5)	8(47.1)	2(16.7)
Ef.3	5(17.2)	3(17.6)	2(16.7)
ypTN^a^, n(%)			
ypTXN0	2(6.9)	2(11.8)	0(0.0)
ypT0N0	4(13.8)	2(11.8)	2(16.7)
ypT0N1	1(3.4)	1(5.9)	0(0.0)
ypT1-2N0	4(13.8)	2(11.8)	2(16.7)
ypT1-2N1	4(13.8)	0(0.0)	4(33.4)
ypT1-2N2	8(27.6)	7(41.2)	1(8.3)
ypT3N0	3(10.3)	3(17.6)	0(0.0)
ypT3N2	2(6.9)	0(0.0)	2(16.7)
ypT2N3	1(3.4)	0(0.0)	1(8.3)

a TNM classification of malignant tumors (8th edition)

b Three sleeve lobectomy cases were included

c *epidermal growth factor receptor*

d *c-ros oncogene 1*

e programmed cell death ligand 1

### Induction therapy

The details of the preoperative induction therapies were as follows: 20 received cisplatin + S-1 + radiation therapy, 3 received carboplatin + paclitaxel + radiation therapy, 3 received other platinum-based doublet + radiation therapy, and 6 received chemotherapy alone. The concurrently administered radiation dose was standardized at 40 Gy, but 1 patient with chest wall involvement received 60 Gy. Only 1 patient developed PD, and the disease control rate (DCR) was 96.9% (Table 1). In the cisplatin + S-1 + radiation therapy group (n = 20), there were 13 patients with a partial response (PR) and 7 patients with stable disease (SD) with an objective response rate (ORR) of 65.0%. The ORR in the other treatment groups was 41.7%, which was slightly lower than that in the cisplatin + S-1 + radiation therapy group. One case of treatment-related death was observed. The details of this patient are provided in the “Adverse events” section. The group that received chemotherapy alone as induction therapy included patients with extensive interstitial pneumonia or widespread metastatic lymph nodes, which made them ineligible for radiation treatment. The group also included cases from a clinical trial involving the administration of immune checkpoint inhibitors during the perioperative period.

### Surgical resection and histological findings

Among the 32 patients, surgical resection was feasible in 29 (90.6%). Among the 29 patients who underwent curative resection, 24 (82.8%) underwent lobectomy, including 3 sleeve lobectomy cases (Table 1). Five patients (17.2%) underwent pneumonectomies. Four of 29 (13.8%) patients required combined resection, including 3 cases of chest wall resection and 1 case of pulmonary artery resection. Complete resection (R0) was performed in 26 (89.7%) patients. Within the subset of 3 R1 cases, 2 patients had malignant pleural effusion and 1 was positive for pleural fluid cytology. In the cisplatin + S-1 + radiation therapy group (n = 20), the rate of progression to surgery was 85%, and complete resection (R0) was achieved in 15 patients (88.2%). Downstaging was achieved in 16 of 32 (50.0%) patients who underwent induction therapy. Among the 17 patients treated with cisplatin + S-1 + radiation followed by surgery, 2 cases, both of which were R1 with intraoperative malignant pleural effusion, started targeted molecular therapy corresponding to their genetic expression of EGFR mutation and ROS1 rearrangement, respectively. Of the remaining 15 patients, 2 did not receive any adjuvant therapy. Ten patients underwent 2 courses of cisplatin plus S-1 therapy, while the remaining 3 received alternative regimens (1 patient received cisplatin plus vinorelbine and 2 were treated with cisplatin + pemetrexed).

Of the 29 patients who underwent surgical resection, 5 (17.2%) showed a complete pathological response (Ef. 3). Moderate histopathological effects (Ef. 2) were observed in 10 patients (34.5%). In the cisplatin + S-1 + radiation therapy group (n = 17), Ef. 3 (complete pathological response) was observed in 3 patients (10.3%). Furthermore, a combined response of Ef. 2 (in which surviving cancer cells were assessed to be less than one-third of the tumor tissue) and Ef. 3 was noted in 11 patients (64.7%).

### Adverse events

Table 2 summarizes the toxicities associated with induction therapy and the postoperative complications. Neutropenia, leukopenia, and constipation were frequently observed as adverse events during induction therapy. Grade 4 adverse events included leukopenia in 1 patient, neutropenia in 2 patients, and febrile neutropenia in 1 patient. All toxicities were manageable, and no cases were deemed inoperable due to these toxicities. Three cases were found to be inoperable due to toxicity of the induction therapy, of which 2 were attributed to radiation pneumonitis. The remaining case involved grade 5 hemorrhagic enterocolitis. The patient completed the first cycle of cisplatin plus S-1 as planned, with grade 2 leukopenia and grade 1 thrombocytopenia observed at the end of the first cycle. However, bone marrow suppression improved before the start of the second cycle of cisplatin + S-1 and no dose reduction was required. The patient completed the second cycle at the regular dose as scheduled, along with the planned 40 Gy of radiation therapy. Nausea, loss of appetite, and fatigue began in the latter part of the second cycle. CT scans revealed circumferential wall thickening throughout the small intestine, while an endoscopic examination showed esophagitis and intestinal ischemia. Concurrently, the patient developed febrile neutropenia and was started on granulocyte colony-stimulating factor (G-CSF) formulations. Despite the lack of improvement in intestinal ischemia and onset of disseminated intravascular coagulation (DIC), comprehensive treatment did not improve the overall condition, leading to death due to hemorrhagic enterocolitis.

**Table 2:** Toxicities of induction therapy(n = 32) and postoperative complications (n = 29)

Induction therapy toxicity*(n = 32)	All	CDDP + S-1 + RT (n = 20)			Others (n = 12)		
	Any Grade	Grade 1–2	Grade 3	Grade 4≦	Grade 1–2	Grade 3	Grade 4≦
Hematologic							
Leukopenia	3	0	0	0	1	1	1
Neutropenia	10	2	2	0	1	3	2
Thrombocytopenia	3	2	0	0	0	1	0
Anemia	0	0	0	0	0	0	0
Non-hematologic							
Febrile neutropenia	4	0	1	1	0	2	0
Anorexia	1	0	0	0	1	0	0
Diarrhea	2	1	1	0	0	0	0
Constipation	4	1	1	0	2	0	0
Depilation	1	0	0	0	1	0	0
Hyperkalemia	1	0	0	0	0	1	0
Venous thrombosis	1	1	0	0	0	0	0
Pneumonitis	3	0	2	0	0	1	0
Esophagitis	3	3	0	0	0	0	0
Hemorrhagic Enterocolitis	1	0	0	1(G5)	0	0	0

Postoperative complication*(n = 29)	All	CDDP + S-1 + RT (n = 17)			Others (n = 12)		
	Any Grade	Grade 1–2	Grade 3	Grade 4≦	Grade 1–2	Grade 3	Grade 4≦
Pulmonary fistula	2	0	1	0	1	0	0
Bronchial fistula	1 1	0	0	0	0 1 0	1	0
Pneumonia	2	0	2	0	0	0	0
Chylothorax	1	1	0	0	0	0	0
Recurrent Nerve Paralysis	2	1	0	0	0	1	0
Empyema	2	0	1	0	0	1	0
ACOS^a^	1	1	0	0	0	0	0

^*^Common Terminology Criteria for Adverse Events v5.0

aAsthma and Chronic obstructive pulmonary disease Overlap Syndrome

The grade ≥ 3 postoperative complications observed in the cisplatin + S-1 + radiation therapy group included 1 case of pulmonary fistula, 2 cases of pneumonia, and 1 case of empyema. In the other treatment groups, complications included 1 case of bronchial fistula, 1 case of recurrent laryngeal nerve paralysis, and 1 case of empyema. No surgery-related deaths occurred. As for adverse events associated with adjuvant therapy, 1 case each of grade 1 pneumothorax, grade 2 late radiation pneumonitis, and grade 3 anorexia was observed; however, no treatment-related deaths occurred during the adjuvant therapy period.

### Survival and recurrence

**Fig. 1:**
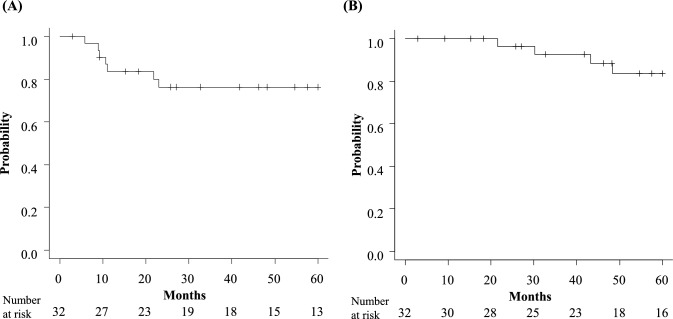
Kaplan–Meier curves for **A** recurrence-free survival and **B** overall survival of patients who underwent induction therapy

**Fig. 2:**
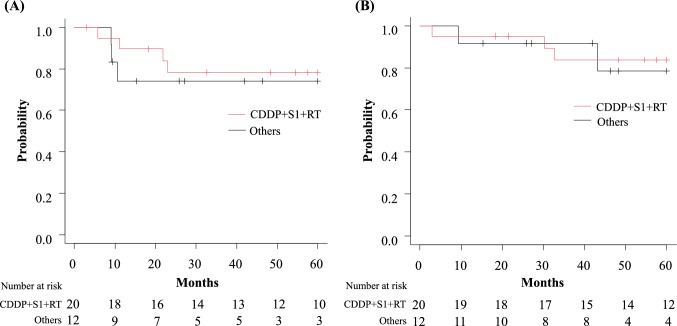
Kaplan–Meier curves for **A** recurrence-free survival and **B** overall survival of patients treated with cisplatin + S-1 + radiation therapy and those who received other induction therapies

The median follow-up duration was 54 months. For all 32 patients who underwent induction therapy, the 3- and 5-year recurrence-free survival (RFS) rates were 76.4% (95% confidence interval [CI]: 56.6–88.0) (Fig. 1A). The 3- and 5 year OS rates of all 32 patients were 86.2% (95% CI: 67.2–94.6) and 82.3% (95% CI: 62.4–92.3), respectively (Fig. 1B). Among the 29 patients who underwent resection, recurrence was observed in 8 patients. In 7 of these patients, the initial site of recurrence was identified in distant regions, with the brain being the most common site of relapse (n = 4). Notably, 1 of the 5 patients who achieved a pathological complete response (Ef. 3) also experienced brain recurrence. Among 17 patients who received cisplatin + S-1 + radiation therapy and underwent surgery, recurrence was identified in 5 cases, including 1 instance of local recurrence and 4 instances of distant metastasis (1 case each of multiple lung metastases and brain, kidney, and choroid involvement). The three-year RFS rate for patients treated with cisplatin + S-1 + radiation therapy (n = 20) was 78.3% (95% CI: 51.9–91.3), and for the 12 patients that received induction therapies other than cisplatin + S-1 + radiation therapy, the rate was 74.1% (95% CI: 39.1–90.9) (Fig. 2A). The three-year OS rate for the cisplatin + S-1 + radiation therapy group was 83.8% (95% CI: 57.7–94.5), and that for the other induction therapy group was 91.7% (95% CI: 53.9–98.8) (Fig. 2B).

Regarding the relationship between clinical factors (histology, N status, down staging) and outcomes, the non-adenocarcinoma group demonstrated a tendency for superior recurrence-free survival rates in comparison to the adenocarcinoma group (5-year RFS rate, 86.2% vs. 62.9%; *p* = 0.13). Additionally, the cN2 group showed a tendency towards a higher recurrence rate than the cN0-N1 group (5 year RFS rate, 58.8% vs. 100.0%; p = 0.09). When patients were categorized based on downstaging versus non-downstaging and pathological response (Ef. 3 vs. Ef. 1–2), no statistically significant differences were observed in RFS. In 17 of the 29 patients who underwent surgery after preoperative therapy, excluding 12 patients who were not tested for PD-L1, the 5-year RFS was 100% in the high expression group (n = 4, PD-L1 ≥ 50%), and the relapse rate tended to be lower than that in the low expression group (n = 13, PD-L1 < 50%) (0% vs. 43.4%; *p* = 0.08). Within the context of the EGFR mutation status, 3 patients who were positive for EGFR mutations experienced recurrence, yet all achieved 5-year survival. Overall survival rates showed no significant differences across any of the clinical factors (Table 3).

**Table 3:** Clinical factors and outcomes of all patients who received induction therapy (n = 29)

Factor	Categories	n	5-year RFS(%)	p- value	5-year OS(%)	p- value
Histology	Non-adenocarcinoma	15	86.2	0.13	84.8	0.61
	Adenocarcinoma	14	62.9		90.9	
N status	cN0-N1	12	100.0	0.09	91.7	0.91
	cN2	17	58.8		86.7	
Downstaging	Downstaging	13	83.9	0.57	92.3	0.60
	No downstaging	16	67.5		82.5	
PD-L1 expression	High expression (≥ 50%)	4	100.0	0.08	100.0	0.61
	Low expression (< 50%)	13	56.6		82.1	
EGFR mutation Status	Positive	4	N/A	0.03	100.0	0.63
	Negative	19	78.9		88.5	
Objective response	PR^a^	16	67.7	0.18	79.3	0.13
	SD^b^ or PD^c^	13	83.1		100.0	
Histologic response	Ef.3	5	N/A	0.95	80.0	0.37
	Ef.1- Ef.2	24	74.6		89.7	

a Partial response

b Stable disease

c Progressive disease

Among the 17 patients who received cisplatin + S-1 + radiation therapy and underwent surgery, there were no cases of recurrence in the non-adenocarcinoma subgroup (n = 6), which suggests a trend toward a more favorable outcome relative to the adenocarcinoma group (5 year RFS: 100% vs. 61.4%; *p* = 0.07). The OS rates showed no significant differences across any of the clinical factors (Table 4).

**Table 4:** Clinical factors and outcomes in the cisplatin + S-1 + radiation therapy group (A) and other group (B). cisplatin + S-1 + radiation therapy group (n = 17)

Factor	Categories	n	5-year RFS(%)	p- value	5-year OS(%)	p- value
Histology	Non-adenocarcinoma	6	100.0	0.07	100.0	0.41
	Adenocarcinoma	11	61.4		88.9	
N status	cN0-N1	6	100.0	0.45	100.0	0.48
	cN2	11	63.6		90.0	
Downstaging	Downstaging	9	88.9	0.37	100.0	0.22
	No downstaging	8	60.0		83.3	
PD-L1 expression	High expression (≥ 50%)	1	N/A	0.29	N/A	0.70
	Low expression (< 50%)	8	50.0		85.7	
EGFR mutation Status	Positive	2	N/A	< 0.01	100.0	0.76
	Negative	12	82.5		90.9	
Objective response	PR^a^	11	72.7	0.40	90.0	0.48
	SD^b^ or PD^c^	6	80.0		100.0	
Histologic response	Ef.3	3	N/A	0.37	N/A	0.70
	Ef.1- Ef.2	14	71.4		92.3	

Factor	Categories	n	5-year RFS(%)	p- value	5-year OS(%)	p- value
Histology	Non-adenocarcinoma	8	85.7	0.14	87.5	0.73
	Adenocarcinoma	4	50.0		66.7	
N status	cN0-N1	6	100.0	0.07	83.3	0.75
	cN2	6	50.0		90.0	
Downstaging	Downstaging	4	75.0	0.74	75.0	0.56
	No downstaging	8	75.0		80.0	
PD-L1 expression	High expression (≥ 50%)	3	100.0	0.29	100.0	0.77
	Low expression (< 50%)	5	N/A		N/A	
EGFR mutation Status	Positive	2	N/A	0.55	100.0	0.31
	Negative	6	80.0		55.6	
Objective response	PR^a^	5	53.3	0.18	53.3	0.10
	SD^b^ or PD^c^	7	85.7		100.0	
Histologic response	Ef.3	2	N/A	0.03	50.0	0.18
	Ef.1- Ef.2	10	80.0		83.3	

a Partial response

b Stable disease

c Progressive disease

## Discussion

The high recurrence rate and poor prognosis of patients with locally advanced NSCLC remain a challenging issue. In the current study, which mainly investigated cisplatin + S-1 + radiation therapy, we observed notably high 3-year OS and RFS rates of 83.8% and 78.3%, respectively, even in inoperable cases**.** Table 5 summarizes the treatment outcomes of neoadjuvant chemoradiotherapy, including outcomes in our own population, for locally advanced NSCLC. The concurrent radiation doses were generally approximately 45 Gy. The ORRs of cisplatin + S-1 + radiation therapy were 35–86% [12, 19, 20]. In the present study, the ORR among the 20 patients treated with cisplatin + S-1 + radiation therapy was 65% and there were no PD cases. The ORR was equivalent to that reported in previous reports using other chemotherapy regimens [2, 3, 21–24].

**Table 5:** Summary of previous clinical trials on neoadjuvant chemoradiotherapy in resectable stage III non-small cell lung cancer

Author	Year	n	Stage	Histology	Regimen	Radiation dose (Gy)	ORR (%)	Surgery (%)	R0 (%)	pCR (%)	OS (%)	DFS (%)	Local recurrence (%)	Distant recurrence (%)
Thomas [21]	2008	264	IIIA-IIIB	Ad:42% Sq:3%	CDDP + ETP	45	50	54	69	N/A	5-year: 21	5-year:16	34	49
Albain[2]	2009	202	IIIA	Ad:39% Sq:32%	CDDP + ETP	45	N/A	87.6	71.3	N/A	5-year: 27.2	5-year: 22.4	10	37
Girard [22]	2010	32	IIIA	Ad:38% Sq:50%	CDDP + VNR or CBDCA + PTX	46	84	100	78	N/A	3-year: 40	3-year: 30	34	38
Katakami [4]	2012	31	IIIA	Ad:74% Sq:16%	CBDCA + DTX	40	25	89.7	N/A	N/A	3-year: 51.7	3-year: 34.5	21	63
Shien[23]	2012	36	IIIA	Ad:47% Sq:47%	CDDP + DTX	20–50	69	100	100	53	5-year: 78.9	N/A	3	8
Yamaguchi[19]	2013	42	IIIA-IIIB	Ad:50% Sq:29%	CDDP + S-1	40	62	93	100	23	5-year: 77.4	5-year: 52	N/A	41
Darling[24]	2015	104	IIIA	Ad:63% Sq:22%	CDDP + ETP	45–70	N/A	N/A	N/A	N/A	N/A (Median:4.2 years)	N/A	20	42
Pless[3]	2015	117	IIIA	Ad:44% Sq:36%	CDDP + DTX	44	61	85	91	N/A	N/A (Median:37.1 months)	N/A (Median:12.8 months)	15	N/A
Tsuchiya[20]	2018	20	IIIA-IIIB	Ad:50% nonAd:50%	CDDP + S-1	40	35	87	100	35	3-year: 58.1	3-year: 52	N/A	N/A
Takamochi [12]	2022	43	IIIA	Sq	CDDP + S-1	45	86	91	79	39	2-year: 70	2-year: 67	15	8
This study	2024	20	IIB-IIIB	Ad:55% Sq:30%	CDDP + S-1	40	65	85	88	18	3-year: 83.8	3-year: 78.3	6	24

*N/A* Not available, *ORR* Objective response rate, *pCR* Pathological complete response, *OS* Overall survival, *DFS* Disease-free survival, *Ad* Adenocarcinoma, *Sq* Squamous cell carcinoma, *CDDP* Cisplatin, *CBDCA* Carboplatin, *ETP* Etoposide, *VNR* Vinorelbine, *PTX* Paclitaxel, *DTX* Docetaxel, S-1 Tegafur/gimeracil/oteracil potassium

In the present study, the surgery rate in the cisplatin + S-1 + radiation group was 85%, which is comparable to that reported in other studies. The surgery rate suggests the safety of this therapy. Notably, one grade 5 adverse event (hemorrhagic enterocolitis) was reported; however, aside from this, all adverse events were grade ≤ 3, indicating an acceptable toxicity profile of cisplatin + S-1 + radiation therapy. Approximately 90% of the patients in this study achieved R0 resection. Although reports from the 2000s indicated somewhat lower rates of R0 resection, ranging from 60 to 70% [2, 21, 22], those after 2010 demonstrated higher rates of R0 completion [3, 4, 12, 19, 20, 23, 24]. This trend may reflect improvements in the chemotherapy and radiotherapy techniques.

In patients with resectable NSCLC, the pCR rate of neoadjuvant chemoradiotherapy has been reported to be associated with significant improvements in event-free survival (EFS) and OS [25]. A pCR serves as a valuable indicator of the efficacy of chemoradiotherapy. The pCR rate for preoperative chemoradiotherapy with cisplatin + S-1 + radiation therapy at our institution was 18%. We chose upfront surgery for N1 cases or single N2 cases that were completely resectable, whereas we generally performed preoperative chemoradiotherapy for multiple N2 cases. This selection bias may have resulted in slightly lower outcomes than those reported in other studies. In this study, the recurrence rate was significantly lower, especially in non-adenocarcinoma cases, and no recurrence was observed. In cases of cStage III B (T4N2M0 or T3N2M0), definitive chemoradiotherapy is considered alongside surgery following preoperative induction therapy. The choice between these approaches is comprehensively determined through discussions with a multidisciplinary team that includes thoracic surgeons, pulmonologists, radiologists, and pathologists. The decision was based on factors such as the extent of the primary tumor, hilar/mediastinal lymph node involvement, and the patient's overall health status. Induction therapy is primarily conducted in cases in which radical resection is deemed possible based on preoperative imaging evaluations. This approach aims to reduce the size of the primary tumor and lymph nodes, potentially avoiding highly invasive surgical procedures, such as pneumonectomy or vascular/tracheobronchial reconstruction. For cases deemed unresectable during preoperative imaging evaluations, including those with infiltrative N2 nodes or T4 disease involving major vascular or tracheal invasion, definitive chemoradiotherapy was selected.

Takamochi et al*.* [12] highlighted the benefits of preoperative cisplatin + S-1 + radiation therapy, particularly for squamous cell carcinoma. This study showed a pCR rate of 39%, showing a high anti-tumor effect, even though it was limited to squamous cell carcinoma. Previous reports have indicated that preoperative chemoradiotherapy yields higher pCR rates in squamous cell carcinoma than in adenocarcinoma [26]. Furthermore, a significantly higher incidence of brain metastatic recurrence after chemoradiotherapy has been reported in patients with advanced stage IIIB non-squamous NSCLC [27]. Additionally, there are findings that suggest that patients with squamous cell histology exhibit higher pCR rates following preoperative chemotherapy or preoperative chemoimmunotherapy than those with other histological types [28, 29]. These insights imply that squamous cell carcinoma may be more amenable to favorable outcomes with preoperative treatment. In the present study, the distant recurrence rate in the squamous cell carcinoma group treated with cisplatin + S-1 + radiation therapy was notably higher than that reported in other trials. These findings suggest the potential merits of conducting future large-scale trials to explore the clinical advantages of preoperative chemoradiotherapy with stratification by histological subtype.

In advanced NSCLC, the brain is cited as one of the most frequent sites for distant metastatic recurrence, with previous reports indicating that the rate of brain metastatic recurrence after induction chemoradiotherapy followed by surgery in stage IIIA NSCLC ranges from 24 to 40% [30–32]. It is believed that the high recurrence rate is linked to undetectable potential micrometastatic cancer cells that are already present throughout the body at the time of surgery [33], and that the blood–brain barrier plays a role in preventing chemotherapy agents from reaching the brain. Achieving a high pCR rate through preoperative chemoradiotherapy has been suggested to lead to lower recurrence rates and a better prognosis [34, 35]. Enhancing the pCR rate might be the key to controlling distant metastatic recurrence of the tumor. From this perspective, it is important to incorporate local treatment as much as possible in the preoperative stage. Mamon et al. reported a tendency for reduced postoperative brain metastatic recurrence in advanced NSCLC surgical cases by adding local radiotherapy not only postoperatively but also preoperatively, suggesting the efficacy of incorporating radiotherapy into the preoperative induction therapy. In our study, the rate of distant metastatic recurrence was 24% in the cisplatin + S-1 + radiation group, with only 1 case (6%) showing brain metastasis. Relative to previous reports, these data are competitive, suggesting that cisplatin + S-1 + radiation therapy could be an effective treatment strategy for controlling micrometastatic distant lesions.

Recently, preoperative chemoimmunotherapy has emerged as a topic of interest. The CheckMate 816 study, focusing on clinical stages IB–IIIA, reported a pCR rate of 24% and a median EFS of 31.6 months with preoperative nivolumab combined with chemotherapy [36]. The KEYNOTE-671 study, involving clinical stage II-IIIB patients, investigated preoperative pembrolizumab with chemotherapy and postoperative pembrolizumab, and reported a pCR rate of 18%, a 2-year EFS rate of 62.4%, and a two-year OS rate of 80.9% [37]. The AEGEAN study for clinical stage II-IIIB patients, which investigated preoperative atezolizumab plus chemotherapy followed by postoperative atezolizumab, reported a pCR rate of 17.2% and a 1-year EFS rate of 73.4%, indicating favorable outcomes [38]. A retrospective analysis by Fang et al*.* [39], which enrolled 211 patients who received neoadjuvant immunotherapy plus chemotherapy and underwent complete tumor resection, showed a pCR rate of 37.9%, an ORR rate of 69.2%, and a DCR rate of 97.7%. These data suggest a higher ORR in comparison to the conventional preoperative treatments. Although the KEYNOTE-671 trial demonstrated a significant prolongation of EFS relative to preoperative chemotherapy alone, there was no significant difference in OS. There are also concerns regarding long-term immune-related adverse events due to the addition of postoperative immunotherapy. Data on perioperative chemoimmunotherapy are still immature and require further long-term follow-up. In addition, no clinical trials have compared perioperative chemoimmunotherapy with induction chemoradiotherapy for resectable locally advanced NSCLC, and future large comparative trials are expected. Although preoperative treatment modalities have changed dramatically in recent years with the development of immune checkpoint inhibitors, there was little change during the study period from 2012 to 2022, and no significant alterations in the type or indications of induction treatments at our institution were observed. However, we believe that induction chemoradiotherapy will remain an important option for patients who are intolerant to immune checkpoint inhibitors or for those who are unlikely to respond to such treatments. As mentioned in the Introduction, induction chemoradiotherapy includes local treatment through irradiation, and when combined with S-1, it enhances tumor radiosensitivity, resulting in a high therapeutic effect. This approach is a promising preoperative treatment option that not only suppresses local recurrence, but also prevents micrometastasis, with the potential for curative outcomes.

The present study was associated with several limitations. This study was a retrospective analysis with a relatively small sample size. The evaluation of lymph node metastasis was not based solely on preoperative biopsies of the hilar or mediastinal lymph nodes but also included a radiographic diagnosis. The inclusion of clinical stage IIB cases may have affected recurrence and/or the prognosis, as these were cases with a lower oncological stage. As mentioned previously, there may also be a selection bias regarding surgical indications.

In conclusion, cisplatin + S-1 + radiation therapy for resectable locally advanced NSCLC can be considered a viable preoperative treatment option owing to its tolerability and efficacy.
